# Global Trends of Lipid Metabolism Research in Epigenetics Field: A Bibliometric Analysis from 2012–2021

**DOI:** 10.3390/ijerph20032382

**Published:** 2023-01-29

**Authors:** Hanqi Liu, Yanqing Huang, Shanshan Lu, Didi Yuan, Junwen Liu

**Affiliations:** Department of Histology and Embryology, Xiangya School of Medicine, Central South University, Changsha 410013, China

**Keywords:** lipid metabolism, epigenetics, bibliometrics, CiteSpace, VOSviewer

## Abstract

Most common diseases are characterized by metabolic changes, among which lipid metabolism is a hotspot. Numerous studies have demonstrated a strong correlation between epigenetics and lipid metabolism. This study of publications on the epigenetics of lipid metabolism searched in the Web of Science Core Collection from 2012 to 2022, and a total of 3685 publications were retrieved. Much of our work focused on collecting the data of annual outputs, high-yielding countries and authors, vital journals, keywords and citations for qualitative and quantitative analysis. In the past decade, the overall number of publications has shown an upward trend. China (1382, 26.69%), the United States (1049, 20.26%) and Italy (206, 3.98%) were the main contributors of outputs. The Chinese Academy of Sciences and Yale University were significant potential cooperation institutions. Articles were mainly published in the “International Journal of Molecular Sciences”. In addition to typical liver-related diseases, “ferroptosis”, “diabetes” and “atherosclerosis” were identified as potential research topics. “NF-κB” and “oxidative stress” were referred to frequently in publications. METTL3 and ALKBH5 were the most discussed m^6^A-related enzymes in 2022. Our study revealed research hotspots and new trends in the epigenetics of lipid metabolism, hoping to provide significant information and inspiration for researchers to further explore new directions.

## 1. Introduction

Lipids are significant substances in a human’s basic physiological activities, which play a vital part in energy storage and metabolism and also act as the second messengers to transmit extracellular stimulus signals. Cholesterol and phospholipids are involved in biofilm formation [1]. Lipid metabolism delivers lipids to peripheral tissues or to return lipids to the liver, which participate in biosynthesis, storage and degradation [2]. The regulation of lipid metabolism, such as lipid uptake, synthesis and hydrolysis, is essential for maintaining cellular homeostasis. The progressive states of most common diseases such as death-heart disease, stroke, diabetes and especially cancer [3] are characterized by metabolic changes that lead to tissue dysfunction [4] and are closely related to the supply of energy. Lipid metabolism can provide the available nutrients for malignant tumor cells or other disease-pertinent cells to support their rapid proliferation, survival, migration, invasion and metastasis. Numerous studies have highlighted the key role of lipid metabolism in diseases, thus, targeting lipid metabolism regulation can effectively control the deteriorating cellular physiological state and alleviate the occurrence and development of diseases [5,6].

Conrad Waddington first established the epigenetics research group in 1965, putting forward the new concept of epigenetics, and related research began to develop gradually. As a branch of genetics, epigenetics studies the heritable changes in gene expression without changes in the nucleotide sequence of genes [7]. Interestingly, metabolism and epigenetics are interrelated. Metabolism can regulate epigenetics through metabolites, and epigenetics can regulate metabolism by regulating the expression of metabolic genes. Numerous existing studies overwhelmingly support that epigenetic mechanisms play important roles in the lipid metabolism involved in many diseases [8,9,10]. 

Bibliometric analysis is a powerful method to visually calculate the academic achievements’ impact and estimate their scientific relevance in various disciplines [11]. Despite methodological limitations, it is still frequently used to intuitively quantify the academic influence of achievements in multiple fields [12]. It typically gathers and analyzes data of existing publications extracted from a database, then assesses current research performance. Bibliometric analysis can help us to better understand how epigenetic mechanisms function in lipid metabolism, then summarize and further evaluate existing research results over the past decade, as well as assist researchers to discover more mechanisms that may influence the lipid metabolic process in an epigenetic way.

Due to the wide range of epigenetic mechanisms, the number of publications from the earliest research to now is too large, and archaic literature may also affect our judgment of recent hot topics. Thus, we tried to perform an analysis on the recent publications related to the epigenetic mechanisms of lipid metabolism. It was in 2012 when methylated RNA immunoprecipitation sequencing (MeRIP-seq) first revealed the m^6^A modification profile in the entire human transcriptome [13]. As a remarkable methylation modification, m^6^A has begun to enter people’s vision and has gradually become a frontier hotspot in the field of epigenetics, leading to the number of epigenetics-related publications significantly surging [14,15]. Now RNA methylation modification has become a vital part of promoting the research of the epigenetic mechanisms in various diseases. Therefore, we decided on “2012” being the starting year of retrieval, which can not only help us to better analyze recent research hotspots but also include emerging fields such as RNA methylation modification to make our research analysis more comprehensive.

Though there have already been lots of publications on the basic research of lipid metabolism, the literature related to lipid metabolism lacks an overall analysis and a systematic description in the epigenetics field. We tried to retrieve studies with the search formula “((TS = (Lipid Metabolism)) AND TS = (epigenetic OR epigenetics)) AND TS = (bibliometric OR bibliometrics)” in the Web of Science Core Collection (WoSCC), but no relevant literature was retrieved. Despite the fact that some bibliometric publications on lipid metabolism in the epigenetics field may be selected through other rare professional words or not utilizing words such as “bibliometric(s)” in titles or abstracts, leading to them not being retrieved, this result still demonstrated that few bibliometric analyses have been performed in this direction.

In this manuscript, to better assist researchers in selecting appropriate journals, collaborators and research directions, we performed our analysis on annual publication outputs, countries, institutions, journals, authors, keywords and citations. Then VOSviewer, CiteSpace, R version 4.2.1, excel and a bibliometric website were utilized to map the hotspots and frontiers of epigenetics in lipid metabolism. In this manner, our analysis revealed the research pattern, can guide future research and can provide new inspiration for researchers in the field.

## 2. Materials and Methods

### 2.1. Data Source and Search Strategy

Relevant subject terms for “Lipid Metabolism” were firstly searched at NCBI (https://www.ncbi.nlm.nih.gov/mesh) (accessed on 28 November 2022) to minimize misses. Then literature retrieval was performed online through the WoSCC (https://www.webofscience.com) on 28 November 2022. In order to involve all relevant literature to the greatest extent possible, in the sub-databases of WoSCC, the Science Citation Index Expanded (SCI-EXPANDED), Social Sciences Citation Index (SSCI), Arts & Humanities Citation Index (AHCI), Emerging Sources Citation Index (ESCI), Current Chemical Reactions (CCR-EXPANDED) and Index Chemicus (IC) were all selected for retrieval. To avoid possible bias on account of daily database updating, we performed all searches within the same day. 

**Figure 1:**
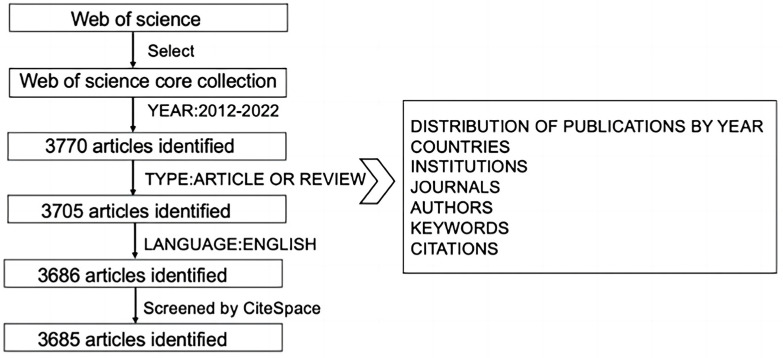
Flow diagram of search strategy in the screening of publications.

Epigenetics mainly includes two categories. One is the regulation of selective gene transcription, including DNA methylation, gene imprinting, histone covalent modification and chromatin remodeling. The other is post-transcriptional regulation of genes, including non-coding RNA, microRNA, antisense RNA, mRNA-related modifications and riboswitches. In order to obtain a comprehensive and accurate retrieved result, we tried to describe research directions in as much detail as possible. The research terms were as follows: ((TS = (Lipid Metabolism OR Lipogenesis OR Lipolysis OR Lipid Mobilization OR Lipoylation)) AND TS = (epigenetic OR epigenetics OR DNA methylation OR genomic imprinting OR chromatin remodeling OR histone modification OR histone acetylation OR histone phosphorylation OR histone glycosylation OR ubiquitination of histone OR histone methylation OR non-coding RNA OR lncRNA OR antisense RNA OR microRNA OR miRNA OR pri-miRNAs OR tiny RNA OR RNA modification OR RNA methylation OR RNA hydroxymethylation OR RNA acetylation OR riboswitch)) refined by WEB OF SCIENCE INDEX (Web of Science Core Collection. SCI) AND DOCUMENT TYPES (ARTICLE OR REVIEW) AND LANGUAGES (ENGLISH), and the timespan of 2012–2022. We then utilized CiteSpace to remove duplicate literature from the retrieved results. The screening and analyzing process is illustrated in Figure 1.

### 2.2. Data Collection

Raw data from the WoSCC database were initially downloaded and verified. Duplicates were removed from the extracted data by CiteSpace for data cleaning. Then the data were imported into Microsoft Excel (Version 16.69.1), Bibliometric online analysis platform (https://bibliometric.com) (accessed on 28 November 2022), VOSviewer (Version 1.6.18; https://www.vosviewer.com) (accessed on 28 November 2022) and CiteSpace VI (Version 6.1 R2; https://CiteSpace.podia.com) (accessed on 28 November 2022) for systematic analyzing.

### 2.3. Statistical Methods

We performed analysis on publications included by annual outputs, countries, institutions, journals, authors, keywords and citations, and tried to extract their characteristics to obtain descriptive results. CiteSpace VI was then utilized for constructing a correlation map in countries’ cooperation and clustering of keywords, and VOSviewer software for network visualizations in some cases such as institutions, authors and keywords. Moreover, CiteSpace VI was applied to burst detection to investigate research directions and institutions with great research potential. R 4.2.1 was utilized for the visualization of world map pertinent data. 

## 3. Results

### 3.1. Annual Publication Outputs

**Figure 2:**
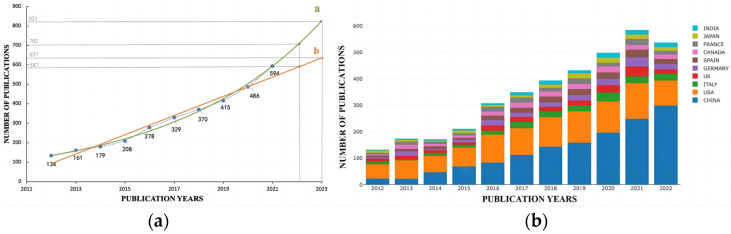
(**a**) Annual publication outputs of publications on epigenetics in lipid metabolism. (**b**) The proportion of articles published by countries each year from 2012 to 2021. (**a**) The number of publications quickly surged year by year from 2012 to 2021. Exponential adjustment (**a**): y = 8E−143e^0.1651x^, R^2^ = 0.9928. Linear adjustment (**b**): y = 49.285x − 99067, R^2^ = 0.9630. (**b**) Different colors represent number of publications of different countries.

We separately counted the number of publications each year in the WoSCC. Overall, a total of 3685 pieces of literature were included in our analysis. During the past decade, publications have risen steadily, showing an upward trend. To justify the law of publication trend, as shown in Figure 2a, exponential and linear fitting were both performed with the extracted data of the annual outputs in the most recent 10 years (2012–2021). The fitting equation of the exponential curve was y = 8E−143e^0.1651x^ (with a correlation coefficient of 0.9928), and the linear fitting equation was y = 49.285x − 99,067 (with a correlation coefficient of 0.9630).

Comparing the two correlation coefficients, the exponential fitting curve was more in line with the trend of the annual publication. By 28 November 2022, the number of pieces of literature in 2022 had reached 531. Given that the publications were consistent with the results of exponential fitting, the total number of publications in 2022 was expected to reach nearly 702 and 821 in 2023. According to the linear fitting, the number of publications in 2022 and 2023 was, respectively, predicted to be 587 and 637. In aggregate, the number of publications has increased rapidly in the past 10 years, which suggests that the role of epigenetic mechanisms in lipid metabolism become more and more of a research hotspot year by year, showing self-evident importance. 

The top 10 countries by the number of publications each year are shown in Figure 2b in different colors, and presents that China and the United States (USA) had absolute dominant positions in terms of their number of publications. The number of publications of the USA was relatively stable, while those of China showed an upward trend year by year. Since 2017, China has surpassed the former and contributed the most to the total publications, and we can also observe upward trends in other countries. Overall, in the past decade, research on epigenetics in lipid metabolism has attracted scientists’ attention and gradually matured.

### 3.2. Global Landscapes of Epigenetics in Lipid Metabolism

**Figure 3:**
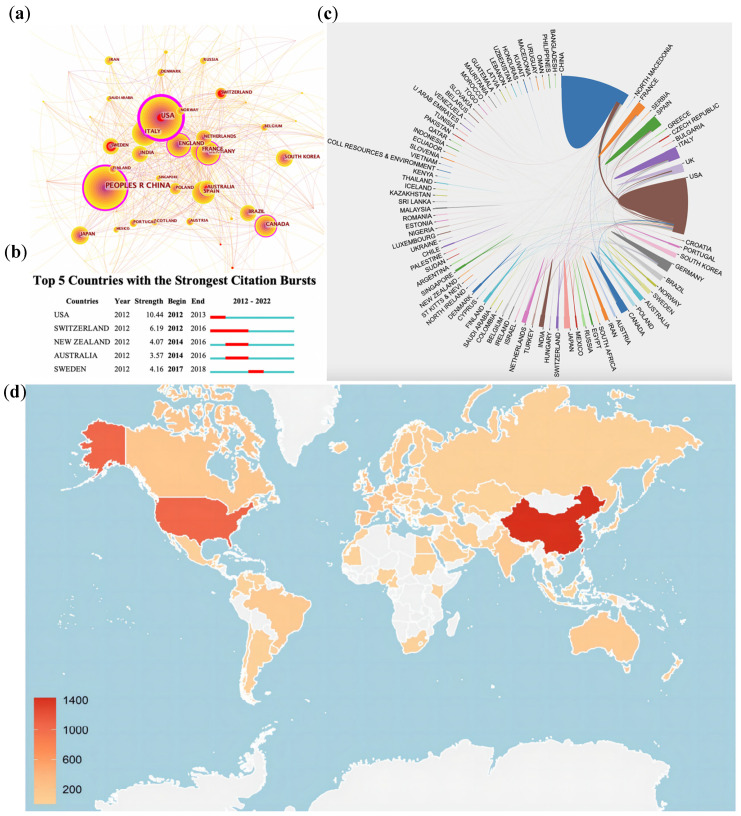
(**a**) Analysis of cooperation between main countries of publication. (**b**) Top 5 countries with the strongest citation bursts. (**c**) Cooperation network among all countries. (**d**) Global map showing countries where the articles were published. (**a**) The size of each node presents its number of documents, and the proportion of the outermost ring means its centrality. Central red circles illustrate nodes’ temporal importance. (**b**) The strongest citation burst means that a variable changes greatly in a short period. Red bars illustrate the duration of the burst.

The 3686 publications on WoSCC were contributed by 94 countries. The top 10 countries by citation counts are shown in Table 1. Our major evaluation indicators were documents, centrality and half-life. The top ranked item by occurrences were China (n = 1382, 26.69%), followed by the USA (n = 1049, 20.26%) and Italy (n = 206, 3.98%). Half-life shows the number of years extrapolated forward from the current time, in which citations accounted for 50% of the total. A longer half-life indicates a more sustained impact of the publications of the country. Extensive collaborations between countries are shown in Figure 3a. The purple circles around nodes represent centrality and indicate the pivotal role in cooperative relationships among countries/regions. A node with high mediating centrality is often a key hub connecting two different domains. The country with the highest centrality was the USA (0.41), followed by China (0.22) and England (0.18). We also analyzed the bursts of each country’s publications, which is show in the form of central red circles in the collaboration network. Bursts indicate the temporal importance of nodes. Detailed information of the top five countries with the strongest citation bursts are shown in Figure 3b. Among them, the USA (10.44) has shown the strongest citation bursts since 2012, but its burst length lasted for only one year, which elucidated that the pertinent research in the USA was rapid and concentrated. Figure 3c,d more intuitively show the cooperation between countries and the outputs of each country. The USA is identified as the center of international cooperation, and there is relatively close collaboration between developed countries.

### 3.3. Major Output Contributors

Over 3966 institutions contributed to the publications on WoSCC. The number of documents, citations, centrality, half-life and total link strength are shown in Table 2. In relation to institutions with the highest number of publications, the Chinese Academy of Sciences (106) leads in terms of output, followed by Zhejiang University (69) and Fudan University (60). Among them, the Chinese Academy of Sciences also has high centrality (0.16), half-life (6.5) and total link strength (157), which shows the output of this institution is vital and meaningful. The top 10 institutions in terms of the number of publications were distributed in China (n = 8), Sweden (n = 1) and the USA (n = 1), indicating that China has a high enthusiasm for epigenetics in lipid metabolism. Further analysis showed that more than 21,539 authors contributed to the writing of 3685 documents. The details of the top 10 authors in terms of the number of publications are shown in Table 3. Fernandez-Hernando, Carlos (27 publications) had the highest number of publications with a high total link strength, followed by Zhao, Ruqian (21 publications) and Loor, Juan J (17 publications). Interestingly, three of the top 10 authors are from Yale University, and Fernandez-Hernando, Carlos, as the most influential author, is also from this institution.

**Figure 4:**
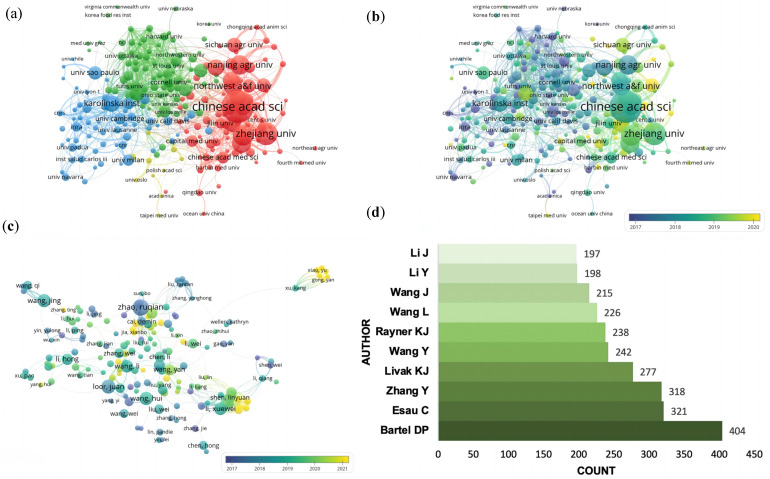
Visualization graphs of contributors. (**a**) Network visualization of institutions involved. (**b**) Overlay visualization of institutions. (**c**) Overlay visualization of authors. (**d**) Top 10 cited authors ranked by citations. (**a**) The main institutions were classified into four clusters, presented by four colors (red, blue, green and yellow). Node size indicates a publications’ number of institutions. The lines between nodes represent the cooperative relationships among organizations, the thickness of which shows the link strength between two nodes. (**b**,**c**) Different colors show the year of publication for each node.

VOSviewer was utilized to construct relationship networks, reflecting the cooperative relationships between research contributors. In total, 226 institutions are classified into four groups, represented by four colors (red, blue, green and yellow) in Figure 4a. The node size indicates the number of publications. The lines between nodes represent the cooperative relationships among institutions, the thickness of which shows the link strength between two nodes and evaluates the degree of cooperation among institutions. The institution correlation time network in Figure 4b shows most of the emerging research organizations appear in the red cluster group. The red cluster is mainly composed of Chinese organizations and is centered with the Chinese Academy of Sciences, indicating that institutions in China are currently devoting more research enthusiasm to the epigenetics of lipid metabolism. Similarly, excluding authors who were not directly related to other, a total of 159 authors were finally utilized to construct the author correlation time network in Figure 4c. We observed that among the three small clusters, two research groups emerged in the past two years. The emergence of the autonomous groups also indicated that more investigators were joining the epigenetics of lipid metabolism research cohort. What is more, the most frequently cited authors in the references of an article can display an author’s contribution in the field and help to find potential partners. The top 10 authors cited in the references are shown in Figure 4d. The top ranked, Bartel, D. P., was cited 404 times, followed by Esau, C (321) and Zhang, Y (318).

### 3.4. Pivotal Journals 

**Figure 5:**
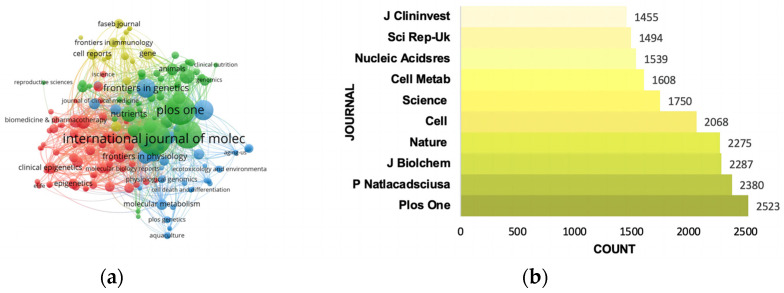
(**a**) Network visualization of institutions. (**b**) Top 10 cited journals ranked by citations. (**a**) The main journals were classified into four clusters, presented by four colors (red, blue, green and yellow). Node size indicates documents of each journal. Lines between nodes represent the connection between journals, the thickness of which shows the link strength between two nodes.

A total of 1040 journals participated in the publication of studies of epigenetics in lipid metabolism from 2012 to 2022 (by 28 November). Details of the top 10 journals by number of publications are listed in Table 4. Impact factor (IF) was utilized to evaluate the significance of a journal (https://clarivate.com/webofsciencegroup/essays/impact-factor/) (accessed on 1 December 2022). Journal Citation Reports (JCR) were the most commonly used international zoning standard, published by Clarivate Analytics, which divided the included journals into 176 different discipline categories, and each discipline category was then divided into four zones according to the impact factor of the journal. Publication in a journal with a higher division indicates that the article is more likely to be of higher quality, and the impact factor can also be used as a criterion for evaluating a publication’s quality. According to the JCR 2021 standards, the IFs of the journals ranged from 3.752 to 17.694, with an average of 6.716, and these journals were mainly distributed in Q1 and Q2. Among the top 10 journals, Nature Communications had the highest IF of 17.694 in 2021. Research results were mainly published in leading journals, indicating that epigenetics in lipid metabolism had a high research value and prospects. The number of publications for the top-three-ranked journals were International Journal of Molecular Sciences (137, 3.72%), Plos One (40, 3.04%) and Scientific Reports (102, 2.77%). The correlations between the involved journals are shown in Figure 5a. As such, it was more likely to find the pertinent references we needed in the above-mentioned journals.

The most frequently cited journals presents the journals in which researchers’ preferred research directions were published, showing their novelty and importance. The top 10 cited journals in references were visualized. According to Figure 5b, citations of the top 10 journals were all above 1455, and the top three journals were, respectively, Plos One (2523), P Natlacadsciusa (2380) and J Biolchem (2287). It is more likely to find valuable research directions in these journals and to inspire scientists to find potential purposeful research.

### 3.5. Trending Topics of Keywords

**Figure 6:**
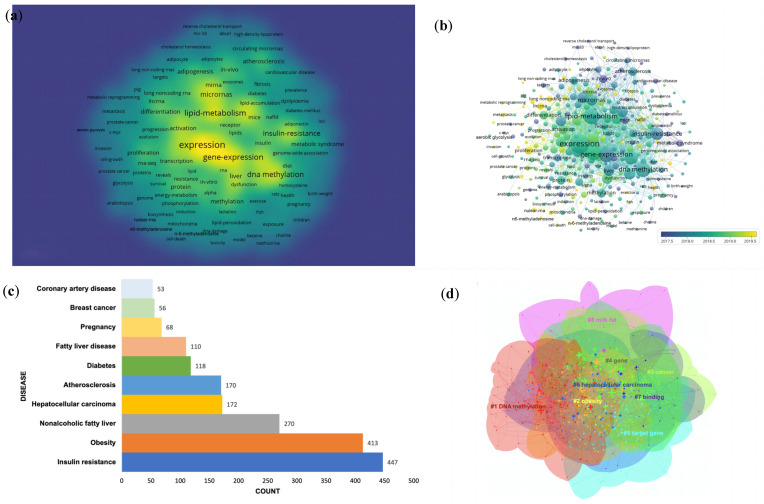
(**a**) Density network visualization of keywords. (**b**) Overlay visualization of keywords. (**c**) Top 10 diseases ranked by occurrences in studies on epigenetics of lipid metabolism. (**d**) Clusters of keywords. (**a**) Higher items’ number near a point, higher adjacent items’ weight, and the color of the point closer to yellow. Conversely, the color of the point is closer to blue. (**b**) Different colors show the year of publication for each node.

To present the knowledge map of keywords for epigenetics in lipid metabolism, a total of 13,913 keywords were extracted from titles and abstracts. VOSviewer was utilized to construct the density and overlay visualization of keywords in Figure 6a,b, which can reflect hot topics visually and show how research hotspots change over time. Taking 20 as the minimum number of occurrences of a keyword, of the 13,913 items, 327 met the threshold. In the density graph, the higher the items’ number is near to a point, the higher the adjacent items’ weight is, and the color of the point is closer to yellow. Conversely, the color of the point is closer to blue. According to the time network, emerging keywords in recent years include N6-methyladenosine, lncRNA and nuclear-RNA, etc., indicating that researchers have begun to shift their focus from DNA methylation to RNA modification. We then analyzed the occurrences of keywords, and the top frequent 10 diseases are shown in Figure 6c. The top three were insulin resistance (447), obesity (413) and nonalcoholic fatty liver (270), respectively. Analysis results also showed that PPAR-γ, miR-122, PPAR-α, SIRT1, miR-33, ABCA1 and P53 showed strong correlations with the epigenetic mechanism of lipid metabolism, and the closely related pathways were NF-κB (enhanced κ-light chain of nuclear factor-activated B cells) and AMPK (Adenosine 5′-monophosphate (AMP)-activated protein kinase). Remarkably, being detected 256 times in keywords, oxidative stress was the physiological state most closely related to epigenetics in lipid metabolism.

**Figure 7:**
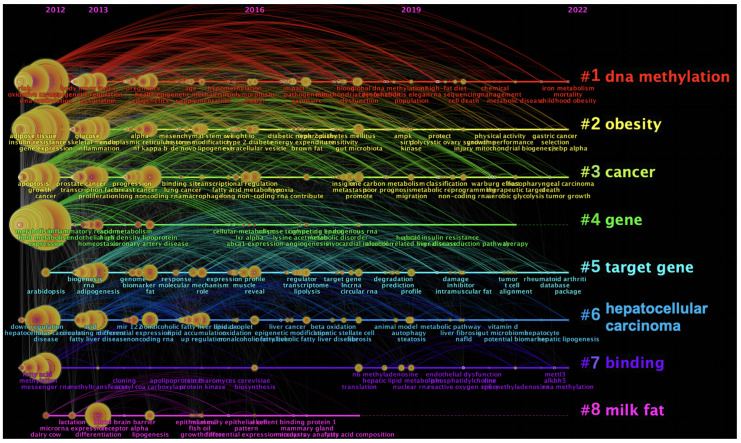
Timeline of keywords. Footnote: Keywords were clustered to different items represented by 8 colors.

CiteSpace was then used to cluster keywords, illustrating the mainstream topics and frontiers in the epigenetics of lipid metabolism pertinent research. As shown in Figure 6d, all the keywords were clustered into seven items, and DNA methylation was identified as one of the major epigenetic mechanisms in lipid metabolism. The change in keywords in each cluster over time are shown as a timeline view in Figure 7. METTL3 and ALKBH5 in #6 binding were the molecules of interest in 2022, which were a significant writer and eraser in m^6^A enzymes. 

**Figure 8:**
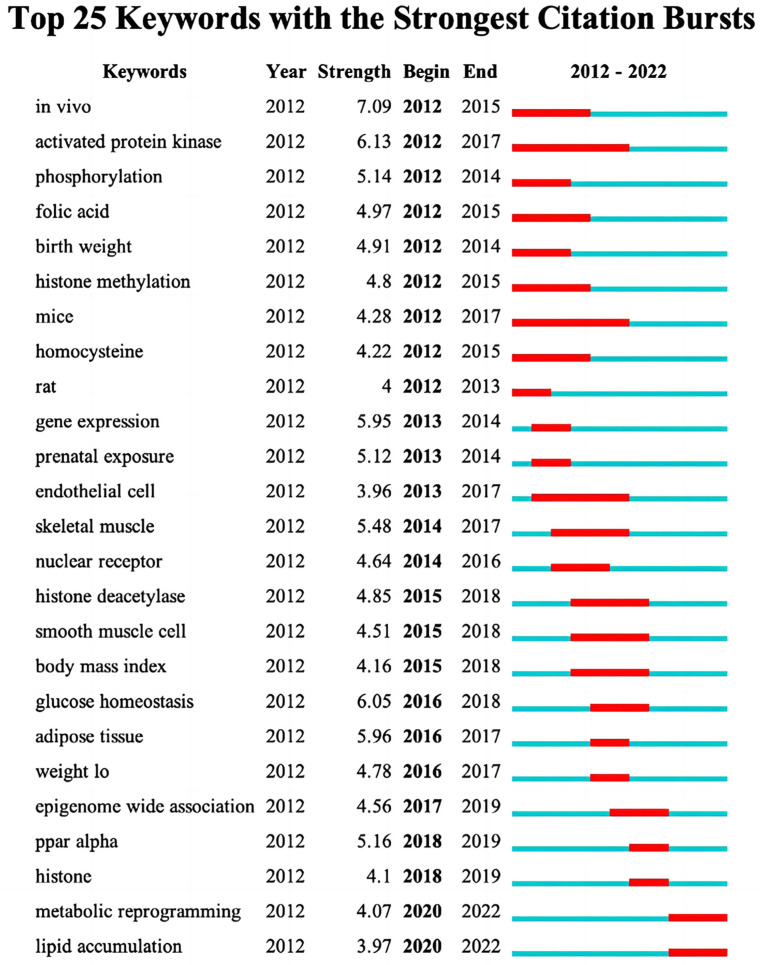
Top 25 keywords with the strongest citation bursts. Footnote: The strongest citation burst means that a variable changes greatly in a short period. Red bars illustrate the duration of the burst.

Burst keywords were detected by CiteSpace, which represents words that have been cited frequently over a period and indicates research frontier topics. Among the extracted keywords, the top 25 entries with obvious outbreak trends are shown in Figure 8. In the past decade, keywords in the early period were mostly related to pregnancy, such as folic acid (2012–2015), birth weight (2012–2014) and prenatal exposure (2013–2014), etc. Then, research on cells became hot topics, such as endothelial cells (2013–2017) and smooth muscle cells (2015–2018), which both showed an explosive trend for a long time. In last two years, “metabolic reprogramming” and “lipid accumulation” have emerged. Metabolic reprogramming refers to cells resisting external environmental stresses by changing metabolic mechanisms and regulating synthetic reactions to counter the energy demands required by different environments. Searching the literature published from 2020 to 2022 involved in metabolic reprogramming, the highly cited literature highlighted the imperative role of lncRNA in metabolic reprogramming [16]. Cellular metabolites served as substrates for epigenetic modification or changing the cellular environment to affect epigenetic modification then further affecting the epigenetic pattern. Conversely, epigenetic modification can also regulate metabolic reprogramming by changing the transcriptional regulation of metabolic enzymes, which is crucial for lipid metabolism [17]. Similarly, during the last two years, the notable research results cited frequently related to lipid accumulation, delineate lncRNA kcnq1ot1, promote lipid accumulation and accelerate atherosclerosis by functioning as a ceRNA through the miR-452-3p/HDAC3/ABCA1 axis [18], and the demethylation of mRNA regulated by FTO can induce lipid accumulation in hepatocytes [19], etc. The above results illustrate that “metabolic reprogramming” and “lipid accumulation” may be major parts that are influenced by epigenetics in lipid metabolism.

### 3.6. Analysis of Citations

Table 5 shows details of the top 10 articles with the highest centrality including the title, journal, first author and year. Cited articles with high centrality were usually important turning points of revolutionary discovery and act as connecting hubs in the research network. Ranked first, “Feedback modulation of cholesterol metabolism by the lipid-responsive non-coding RNA LeXis” [20] was published in Nature by Sallam T et al. The second and third articles in turn were “m^6^A RNA methylation promotes XIST-mediated transcriptional repression” and “Long Noncoding RNA HULC Modulates Abnormal Lipid Metabolism in Hepatoma Cells through an miR-9-Mediated RXRA Signaling Pathway”. Analyzing the journals in which the articles were published, “Nature” appeared more frequently, indicating that pertinent publications in this journal were more favored by scholars. It can be found that most of these articles are closely related to liver lipid metabolism. Half of the top 10 publications focused on lncRNA (n = 5), followed by m^6^A (n = 2), microRNA (n = 2) and DNA (n = 1), illustrating that lncRNA may be at the frontier of epigenetics in lipid metabolism, and researchers can pay more attention to this field.

**Figure 9:**
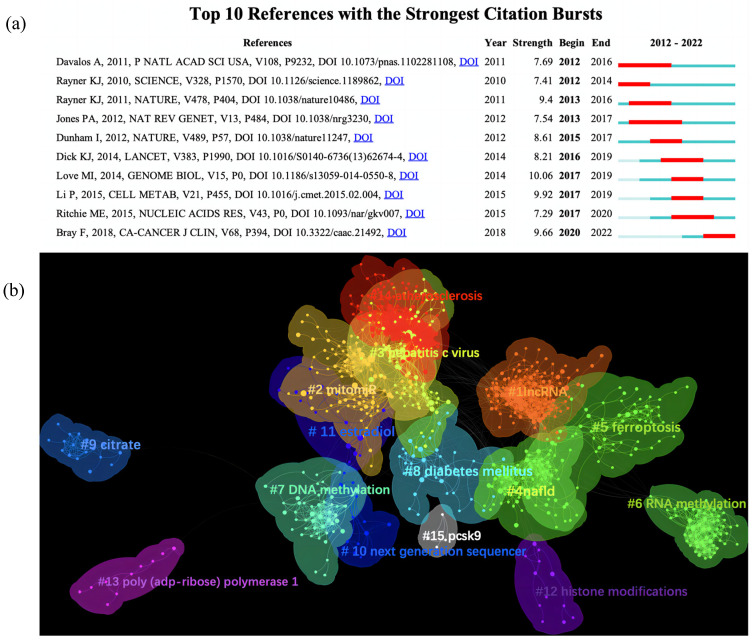
(**a**) Top 10 references with the strongest citation bursts. (**b**) Clusters of reference. The strongest citation burst means that a variable changes greatly in a short period. Red bars illustrate the duration of the burst in (**a**).

CiteSpace was utilized to detect burst references, which represented that articles had been cited frequently over a period and indicated research frontier topics. The top 10 references with the strongest citation bursts are shown in Figure 9a. References are then clustered in Figure 9b based on the log likelihood ratio (LLR) in CiteSpace and a total of 15 clusters were found with a modularity Q of 0.738 and weight mean silhouette S of 0.925. It is generally accepted that Q > 0.3 means a significant cluster structure and S > 0.7 means the clustering is convincing, representing a good homogeneity of our clusters. The 15 clusters summarize 3 main aspects. Firstly, the relevant disease or test involved in epigenetics, including (#3 hepatitis c virus, #4 nafld, #5 ferroptosis, #8 diabetes mellitus, # 10 next generation sequencer, #14 atherosclerosis), followed by the function targets clustered as (#1 lncRNA, #2 mitomiR, #6 RNA methylation, #7 DNA methylation, #12 histone modifications), and finally the epigenetic mechanism such as (#9 citrate, # 11 estradiol, #13 poly (adp-ribose) polymerase 1, #15 pcsk9).

## 4. Discussion

Bibliometric analysis is increasingly being utilized to assess trends and progress in various research fields. As a frontier hotspot in the medical field, epigenetics has been identified as playing an increasingly important role in various diseases’ pathogenesis. The relationship between lipid metabolism and epigenetics has also begun to attract attention. However, to date, there has been no bibliometric analysis of epigenetics in lipid metabolism. This paper used bibliometric analysis to comprehensively analyze the current status and development trend of epigenetics in lipid metabolism from 2012 to 2022 (up to 28 November 2022). Based on inclusion and exclusion criteria, we adjusted the original results of 3770 pertinent papers published in the core journals of Web of Science, and finally screened 3685 articles for inclusion in our analysis. The results showed that the number of publications in this field showed a typical overall upward trend, illustrating this field became more mature and in the spotlight.

China, the United States and Italy were the main countries contributing to the publications. China and the USA outperformed other nations in both the quality and quantity of publications. However, compared with the USA, even with a significant number of publications, China’s centrality was still lower and lacked international cooperation. Developed countries made up 8 of the top 10 and most of the cooperation was only between developed countries, indicating further cross-national collaboration is needed to speed up the research process. Most of the institutions concentrating on the epigenetics of lipid metabolism were from China, and the emerging research groups still belonged to China. The Chinese Academy of Sciences was represented as playing a major role in the sizable group of international institutions. Interestingly, three of the top ten authors were from Yale University, indicating that it was more likely to find influential authors or partners at this institution.

As the primary carrier of research results, journals on the one hand assist in better disseminating publications; on the other hand, they may also serve as indexes to evaluate the quality of research published in them. The top 10 journals with the highest outputs were mostly dispersed in Q1 and Q2, which proved that articles in this field are of high quality and this research direction is of great significance and remarkable. Furthermore, highly published and cited journals help us to better find and select valuable references. The most pertinent articles were published in the International Journal of Molecular Sciences, Plos One and Scientific Reports. Co-cited journals showed that most of the cited references were derived from Plos One. In brief, from these journals, it was more likely to find the useful targeted reference literature we needed.

An analysis of keywords and citations helped us to discover research frontiers and potential topics. High-frequency keyword analysis showed that the top three diseases were liver-related such as insulin resistance, obesity and nonalcoholic fatty liver disease (NAFLD). Lipid metabolism plays a key role in lipid transport between the liver and peripheral tissues, which makes it clear why lipid metabolism disorders are mostly associated with liver diseases. Further combining the analyzed keywords of pertinent publications and references, NAFLD and atherosclerosis were the diseases mainly referred to in both included studies and citations, which not only implies that these diseases have been brought into sharper focus now, but, more importantly, they represent great research prospects in the future. NAFLD is a chronic liver disease worldwide, affecting nearly 24% of the population all over the world. If not controlled in time, NAFLD may even progress to nonalcoholic steatosis hepatitis (NASH) or liver cancer [21]. A growing body of research has also tried to explore epigenetic patterns in the lipid metabolism of NAFLD [20,21]. Cardiovascular disease is the leading cause of mortality worldwide, and atherosclerosis is an important risk factor for this. It is worth noting that lipid accumulation has been identified as the key point in the process of atherosclerosis [22]. Numerous research has proved that epigenetics plays vital roles in atherosclerosis [23]. Therefore, the epigenetics of lipid metabolism in atherosclerosis may also be a significant potential research direction for illustrating the pathogenesis of atherosclerosis.

Our results also represented that PPAR-γ, miR-122, PPAR-α, SIRT1, miR-33, ABCA1 and P53 are, in turn, strongly correlated with the epigenetic mechanisms of lipid metabolism. These most involved target molecules help to point out potential directions for our future research. Researchers could try to focus on the roles of these high-frequency molecules in NAFLD and atherosclerosis, which are more likely to discover the epigenetic mechanisms influenced by these molecules in the lipid metabolic processes of the above-mentioned diseases. In the latest publication in 2023, Tarik Zahr et al. elucidated that PPAR-γ deacetylation can inhibit aging-related atherosclerosis and hypercholesterolemia [24]. PPAR-γ belongs to the nuclear receptor family of activated ligands, mainly expressed in fat cells [25], which explains well why this molecule had the highest frequency among the involved lipid metabolic literature and echoed our reasoning about valuable research directions. Of note, PPAR-α in the same family is also the subject of interest in the epigenetic mechanisms of lipid metabolism. Several PPAR-regulated factors and PPAR regulators performed as epigenetic effectors in many diseases [26], indicating the significance of further exploration about these molecules. Combined analysis of vital authors can also help us to find frontiers of the research. Bartel D. P., who is affiliated with the Howard Hughes Medical Institute, has mainly researched miRNA [27] and has an H-index (Hirsch-index) reaching 105. His research results have been cited many times in references, indicating the lipid metabolism pertinent research in epigenetics is closely related to microRNA. Secondly, Esau, C is affiliated with AptamiR Therapeut Inc. “miR-122 regulation of lipid metabolism revealed by in vivo antisense targeting” published in “Cell metabolism” is his most frequently cited article, the citations of which in all fields have reached 1641. This research implicates miR-122 as being a key regulator of cholesterol and fatty acid metabolism in the adult liver and may be an attractive therapeutic target for metabolic disease [28]. What is more, miR-143 [29] and miR-33 [30] have also been proposed as effective targets related to lipid metabolism in his highly cited literature, and these may also be key molecules that have received much attention from researchers. In addition to atherosclerosis, studies on NAFLD have revealed that these molecules play important roles in the epigenetics of lipid metabolism. Castro, Rui E. et al. demonstrated in their study that acetylated p53 increased with disease severity, and SIRT1 was diminished in the NAFLD liver [31]. Moreover, as a well-known member of the superfamily of ATP-binding cassette, ABCA1 usually functions as a cholesterol efflux pump in a human’s cellular lipid removal pathway. During lipid metabolism in cardiovascular disease, the vascular transcriptional machinery of fundamental genes such as ABCA1 is altered [32]. For example, in some other diseases, ABCA1 has also been identified as a differentially methylated gene in acute coronary syndrome (ACS), and is strongly associated with epigenetic mechanisms in osteoarthritis [33]. In conclusion, further exploration of the above-mentioned epigenetic sensitive biomarkers related to lipid metabolism can make it easier to illustrate the epigenetics in lipid metabolism, and provide new inspiration for the prediction and treatment of some difficult miscellaneous diseases.

Notably, the NF-κB and AMPK pathways are frequently found in keywords. NF-κB is a protein complex that controls transcribed DNA, cytokine production and cell survival, and is also involved in cellular responses to stimuli and the immune response to infection. Existing studies have demonstrated that the NF-κB pathway can regulate lipid metabolism and then affect DSS-induced colitis [34], atherosclerosis [35] and NAFLD [36]. The latest research has disclosed that nicotinamide riboside ameliorate high-fructose-induced lipid metabolism disorder may be related to the regulation of inflammation mediated by the SIRT1/NF-κB signaling pathway [37]. AMPK is also identified as a key molecule in the regulation of biological energy metabolism and lipid metabolic-related diseases [38]. Paying more attention on the above molecules and signaling pathways may be of important research significance to study the possible mechanisms in the epigenetic field of lipid metabolism. Additionally, being detected 256 times among the keywords, oxidative stress was the physiological state in lipid metabolism most closely related to epigenetics. Yosuke Okuno et al. delineated that overexpressing Cat and Sod1 in adipocytes of fat ROS-eliminated mice can exhibit adipose expansion, decrease ectopic lipid accumulation and improve insulin sensitivity, which illustrates that oxidative stress and lipid accumulation are strongly related [39]. In the epigenetics field, up-regulation of FTO expression in NAFLD rats has been demonstrated as being involved in oxidative stress and lipid deposition [40].

According to the timeline of keywords, METTL3 and ALKBH5 were the molecules of interest in 2022, and were vital writers and erasers among the m^6^A enzymes. Based on our above analyzed results, the focus of research on the epigenetic mechanism in lipid metabolism has gradually shifted from DNA methylation to RNA. M^6^A-related enzymes change the methylation of sites on mRNA, which can affect the process of RNA nuclear export, degradation and translation, and then regulate cell fate [41]. In vitro knockdown of METTL3 increased the expression and stability of peroxisome proliferator-activated receptor-α (PPAR-α) mRNA, thereby reducing lipid accumulation [42]. A recent study showed that METTL3 inhibits hepatic insulin sensitivity and promotes fatty acid metabolism by regulating the m^6^A methylation level of fatty acid synthase mRNA [43]. Lipid accumulation was identified as being decreased by knockdown of ALKBH5 in HepaRG and HepG2 cells [44]. The above results all illustrate the critical regulatory role of METTL3/ALKBH5 in lipid metabolism. As a research hotspot in recent years, m^6^A has attracted more and more attention in the field of lipid metabolism. Lipid metabolism mainly affects liver-related diseases; thus, further exploration of the relationship between METTL3/ALKBH5 and lipid metabolism may reveal the key epigenetic mechanisms regulating liver-related diseases.

Citations’ analysis presented the evidence that “lncRNA” was the major theme in the high-frequency co-cited literature. lncRNAs refer to the transcripts > 200 nt in length that do not encode protein [45]. A piece of literature published in 2021 summarized the mechanisms as to how lncRNAs regulate aberrant lipid metabolism in cancer and discussed the mechanism by which lncRNAs affect ferroptosis [46]. This study summarized the current research progress of lncRNAs in the field of lipid metabolism and tried to link ferroptosis with it. Based on this, scientists can open up new research directions and further explore how lncRNAs function in lipid-metabolism-related fields through epigenetic mechanisms. References clusters showed that in addition to liver-related diseases, ferroptosis, diabetes mellitus and atherosclerosis are also notable diseases. Therefore, we reasonably speculate that research focused on these research directions will become potential hotspots in the epigenetics of lipid-metabolism-related study. Putting eyes into these areas will make it more likely to find some valuable new discoveries.

Our study evaluated the trend and status of the epigenetics of lipid metabolism by using bibliometric software such as VOSviewer and CiteSpace, but the results also had some unavoidable limitations. As the bibliometric software were not able to combine two or more databases for analysis, publications involved in our analysis were all abstracted from the Web of Science, which may mean that some pertinent literature was not retrieved. Although we repeatedly searched for the keywords as completely as possible, we still could not avoid losing some articles. In addition, some recent high-quality publications had not appeared for a long time and had low citation frequency, and some others did not utilize commonly used keywords in their titles or abstracts, leading to them not being taken into consideration. These issues were the limitations of this paper. We would try to optimize the selection of the bibliometric methods in the future to include all the publications as completely as possible. What is more, rather than only focusing on highly cited literature, researchers should pay more attention to the latest publications and keep up with the frontier research. Then we could obtain a more comprehensive picture of the research trends in related fields and find new valuable research directions.

## 5. Conclusions

The rapid growth trend of publications on epigenetics in lipid metabolism proves its great research prospects. As far as we are concerned, this paper has published the first bibliometric analysis of trends in the pertinent research on the epigenetics of lipid metabolism. The major contributors and leading journals have been identified in this field. The epigenetic mechanisms related to lipid metabolism mainly affect diseases such as insulin resistance, obesity and NAFLD. In addition to typical liver-related diseases, ferroptosis, diabetes mellitus and atherosclerosis were also detected as significant potential research topics. In addition to NF-κB and AMPK signaling pathways, molecules such as PPAR-γ, miR-122, PPAR-α, SIRT1, miR-33, ABCA1 and P53 have been involved frequently in the literature. Combined detection of the correlation between NAFLD/atherosclerosis and the above molecules is a potential significant direction. Oxidative stress was found to be the physiological state of lipid metabolism most closely related to epigenetics. The research focus of epigenetic mechanisms has shifted from DNA methylation to RNA such as lncRNA and m^6^A, among which METTL3 and ALKBH5 have been shown to be the most studied m^6^A-related enzymes in 2022. In the forthcoming years, researchers could attentively monitor relevant studies to discover further fresh insights.

## Figures and Tables

**Table 1:** The Top 10 countries by documents.

RANK	Country	Documents	% of Total	Centrality	Half-Life
1	China	1382	26.69	0.22	7.5
2	USA	1049	20.26	0.41	5.5
3	Italy	206	3.98	0.09	6.5
4	Germany	193	3.73	0.17	6.5
5	Spain	184	3.55	0.09	6.5
6	England	171	3.30	0.18	6.5
7	Canada	169	3.26	0.12	5.5
8	France	159	3.07	0.09	5.5
9	Japan	125	2.41	0.05	6.5
10	India	114	2.20	0.03	6.5

**Table 2:** Institutions ranked by documents.

Rank	Institutions	Documents	Citations	Centrality	Half-Life	Total Link Strength
1	Chinese Academy of Sciences	106	2987	0.16	6.5	157
2	Zhejiang University	69	1597	0.06	4.5	71
3	Fudan University	60	1375	0.06	4.5	76
4	Nanjing Agricultural University	56	1525	0.02	4.5	30
5	Shanghai Jiao Tong University	55	1334	0.03	3.5	68
6	Northwest A&F University	53	829	0.01	3.5	28
7	Karolinska Institute	46	1405	0.16	3.5	73
8	University of Illinois	46	1084	0.02	4.5	41
9	Chinese Academy of Agricultural Sciences	46	662	0.02	2.5	27
10	University of Chinese Academy of Sciences	45	828	0.05	6.5	81

**Table 3:** Pivotal authors of documents.

Rank	Author	Affiliation	H-Index	Documents	Citations	Total Link Strength
1	Fernandez-Hernando, Carlos	Yale University	61	27	1572	79
2	Zhao, Ruqian	Tianjin Medical University	33	21	443	35
3	Loor, Juan J	University of Illinois Urbana-Champaign	58	17	381	26
4	Wang, Jing	Dalian Maritime University	84	16	185	23
5	Wang, Hui	Jilin University	99	16	160	16
6	Li, Xuewei	Chinese Academy of Sciences	30	15	265	84
7	Wang, Lili	Qingdao University of Science & Technology	116	15	370	10
8	Zhu, Li	Zhejiang University	113	13	239	84
9	Suarez, Yajaira	Yale University	51	13	739	56
10	Goedeke, Leigh	Yale University	27	13	893	54

**Table 4:** Top 10 journals with the largest number of documents.

Rank	Journals	Counts	JCR Partitions	Impact Factor (2021)
1	International Journal of Molecular Sciences	137	Q1	6.208
2	Plos One	112	Q2	3.752
3	Scientific Reports	102	Q2	4.997
4	BMC Genomics	59	Q2	4.558
5	Frontiers In Genetics	52	Q2	4.772
6	Nutrients	36	Q1	6.706
7	Cells	34	Q2	7.666
8	Frontiers In Physiology	32	Q1	4.755
9	Nature Communications	31	Q1	17.694
10	Frontiers In Endocrinology	29	Q1	6.055

The full form of JCR is “journal citation reports”.

**Table 5:** Top 10 highly cited publications.

Rank	Title	Journal	First Author	Year
1	Feedback modulation of cholesterol metabolism by the lipid-responsive non-coding RNA LeXis	Nature	Sallam T	2016
2	m^6^A RNA methylation promotes XIST-mediated transcriptional repression	Nature	Patil DP	2016
3	Long Noncoding RNA HULC Modulates Abnormal Lipid Metabolism in Hepatoma Cells through an miR-9-Mediated RXRA Signaling Pathway	Cancer Res	Cui M	2015
4	Circadian Clock Regulation of Hepatic Lipid Metabolism by Modulation of m^6^A mRNA Methylation	Cell Rep	Zhong X	2018
5	Long noncoding RNAs regulate adipogenesis	P Natl Acad Sci USA	Sun L	2013
6	Genome-wide identification of microRNAs regulating cholesterol and triglyceride homeostasis	Nat Med	Wagschal A	2015
7	miR-27b inhibits LDLR and ABCA1 expression but does not influence plasma and hepatic lipid levels in mice	Atherosclerosis	Goedeke L	2015
8	Identification of a novel human long non-coding RNA that regulates hepatic lipid metabolism by inhibiting SREBP-1c	Int J Biol Sci	Li D	2017
9	Long noncoding RNA MALAT1 promotes hepatic steatosis and insulin resistance by increasing nuclear SREBP-1c protein stability	Sci Rep-UK	Yan CF	2016
10	An integrated encyclopedia of DNA elements in the human genome	Nature	Dunham I	2012

## Data Availability

All data for this study can be found in the WOS (https://www.webofscience.com). The original contributions presented in the study are included in the article. Further inquiries can be directed to the corresponding author.

## Abbreviations

H-index: Hirsch-index; the USA, the United States; WoSCC, Web of Science core collection; IF, impact factor; JCR, journal citation reports; NF-κB, enhanced κ-light chain of nuclear factor-activated B cells; AMPK, Adenosine 5‘-monophosphate (AMP)-activated protein kinase; NAFLD, nonalcoholic fatty liver disease; FTO, fat mass and obesity-associated; PPAR-α/γ, proliferator-activated receptor-α/γ; SIRT1, sirtuin 1; m^6^A, N6-methyladenosine; ABCA1, ATP-binding cassette transporter A1; Tumor Protein P53, P53; non-alcoholic steatosis hepatitis, NASH; METTL3, Methyltransferase 3; ALKBH5, AlkB Homolog 5.
